# Investigation and modeling of odors release from membrane holes on daily overlay in a landfill and its impact on landfill odor control

**DOI:** 10.1007/s11356-020-10793-1

**Published:** 2020-09-17

**Authors:** Jun Jiang, Jianhua Li, Sami Rtimi

**Affiliations:** 1 College of Environmental Science and Engineering, Tongji University, Shanghai, China; 2 Hangzhou Urban Construction Investment Group Co., Ltd., Hangzhou, China; 3 Ecole Polytechnique Fédérale de Lausanne, EPFL-STI-LTP, Station 12, CH-1015 Lausanne, Switzerland

**Keywords:** Landfill site, Odors release, NH_3_, H_2_S, Modeling, Environment

## Abstract

In the present work, we studied the NH_3_ and H_2_S odor fluxes between the exposed working area and the HDPE covering film holes of the daily overlay in an actual landfill site with a daily operating area of 1600 m^2^ in Hangzhou, China. We showed that the odors were released from the membrane pores and the average concentrations of NH3 and H2S release reached 109.6 ± 56.6 and 86.0 ± 31.1 mg/m^2^/s, respectively. These concentrations are 43.8 and 57.3 times the exposed working surface. Furthermore, mathematical modeling based on the total amount of odor release revealed that there was a linear positive correlation between the total odor amount and the landfill operation area. However, the maximum number of film holes allowed on the covering layer has nothing to do with the working area and exposed working time, which is mainly determined by the HDPE film width in terms of ensuring the deodorizing effect of the covering operation. If the HDPE film with a width of more than 4 m is used, the number of film holes allowed within 100 m is more than 8. Therefore, in order to reduce the odor, the appropriate film width should be selected according to the actual operating conditions such as the mechanical operation level at the time of welding, the design of the landfill site, and the operational norms. This study explores the effect of film hole quantity of the daily cover in the landfill on the odor release from the landfill, which can provide an important reference for the design, operation, and decision-making of the daily cover operation of the sanitary landfill.

## Introduction

Odor accounts for less than 1% v/v of total landfill gas (Lim et al. 2018), but because of its harm to surrounding air quality, it affects the health of surrounding residents (Liu et al. 2015; Njoku et al. 2019). Therefore, it has been considered the biggest pollutants to landfills (Drew et al. 2007). Landfill odor control has attracted the attention of landfill managers, designers, and the neighboring areas (Hurst et al. 2005; Njoku et al. 2019).

There are many materials used for temporary covering in landfills, including traditional covering materials such as high-density polyethylene (HDPE) film, geotextiles, and geosynthetic bentonite cushion (GCL) (Querio and Lundell 1992; Capaccioni et al. 2011; Take et al. 2012; Rowe et al. 2016), and some non-traditional materials such as construction waste, waste wood, and other waste (Solan et al. 2010; Hurst et al. 2005). Covering with traditional materials will occupy the storage capacity of the landfill and reduce its space for landfill waste. The use of non-traditional materials, namely waste, as covering materials can effectively improve this problem, so it has become a research hotspot in recent years (Narani et al. 2020; Ait-Benichou et al. 2008; Solan et al. 2010). However, the instability of non-traditional materials and the odor reduction effect still need to be verified.

Daily landfill cover with membrane, construction waste, compost, wood chips, among others can help reducing odor emission (Solan et al. 2010; Hurst et al. 2005; Capaccioni et al. 2011) as well as other nuisances such as windblown litter, vermin, flies, and birds (EPA 1997). Plaza et al. (2007) have shown that overlays can act as effective barriers for gasses and odors. Therefore, the temporary covering operation is widely used as an economical and effective odor control method in the current landfill operation process.

The current landfills are still dominated by traditional daily covering materials, especially high-density polyethylene film due to its low permeability (permeability coefficient can reach 10^−14^ m^2^/s), enough toughness, appropriate weather resistance, good stability, and easy operation (Hwan and Chung 2008; Rowe et al. 2016). HDPE is widely used in daily landfill cover for actual operations.

When using HDPE for landfill day coverage, membrane holes may be generated due to carelessness, errors, or accidents. If the wrong or careless construction method is used on site, the probability of film holes will be greatly increased. For occasional accidents (such as a falling tool), these membrane holes will be few and far apart from each other. Starting from the landfill design phase, the number of membrane holes on site will depend on the measures taken by the project, including material testing, construction, and construction supervision. Of course, it is possible not to produce membrane holes, but this possibility is very small, because it means that no errors occurred and the used construction method is safe, which is impossible in practice (Gilson-Beck 2019). McQuade and Needham (1999) pointed out that the probability of the formation of membrane holes is closely related to the material’s resistance to environmental stress cracking, friction performance, effectiveness of protective materials, and careful manipulation by landfill engineers.

Several studies confirmed that membrane holes will lead to odor leakage. Based on the theoretical derivation, the membrane/geomembrane pore leakage depends on the liner cross section and contact between the geomembrane and the underlying low-permeability layer (Giroud 1997; Rowe 2018). In general, the quantity and size of holes (especially those caused by wrinkles or seams) have a great impact on the geosynthetic clay liner (GCL) leakage (Mendes et al. 2010, Rowe 2012; Fox et al. 2011; El-Zein and Rowe 2008; Rowe and Abdelatty, 2012). However, these studies mostly focused on the leakage of leachate from different anti-seepage systems of the landfill and its impact on the groundwater. Most of the research on landfill focuses on odor concentration, diffusion, deodorization measures, and their impact on the surrounding environment and health (Lim et al. 2018; Liu et al. 2015; Njoku et al. 2019; Cheng et al. 2019).

In this study, we focus our attention on the leakage source of odors mediating the membrane holes of the daily cover layer. It was unclear how the odor leaks on the membrane holes. We also show that the leak will determine whether the temporary covering operation of the landfill is meaningful for odor reduction. This study takes an actual landfill in Hangzhou as the site, to study the effect of film holes on the temporary cover film on the control of landfill odors using NH_3_ and H_2_S as indicators. We aim to (i) clarify the leakage of odors from the film holes from the temporary cover layer and (ii) determine the maximum number of membrane holes allowed to achieve the odor reduction based on mathematical linear modeling. We tried to provide important references for landfill design, operation, and management.

## Material and methods

### Landfill and its operation process

**Fig. 1.**
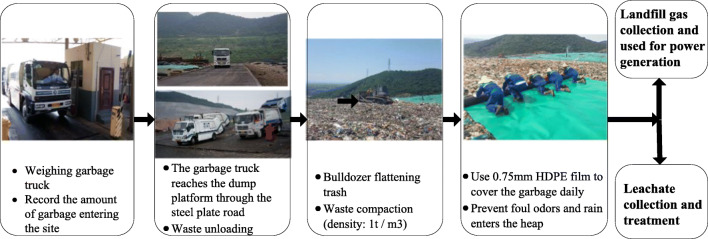
Tianziling Landfill operation method

The landfill station selected in this study is Hangzhou Tianziling Second Landfill, which is in the Qinglongwu Valley (120.2 E, 30.3 N) of Tianziling Mountain in Shitang Village, Banshan Town, northern suburb of Hangzhou. It is a 22.02 million–m^3^ station. It adopts a combination of vertical and horizontal anti-seepage technologies. It is operational since 2007. The landfill receives more than half of the waste in Hangzhou, with an average daily landfill volume of more than 5000 t and a daily maximum landfill volume of more than 6000 t. Hangzhou is the first city in China to pilot garbage cleaning and direct transportation, that is, mixed domestic garbage generated by residents is loaded by garbage trucks and sent directly to a landfill for landfill disposal. There is no garbage transfer station in the middle. The landfill uses the independently established “Tianziling Landfill operation method” to standardize landfill treatment of garbage as shown in Fig. 1.

After the garbage is transported to the landfill by the trucks, the garbage is first weighed, then the truck drives to the reservoir area and arrives the dump platform in the reservoir area by the steel plate road to discharge the garbage. After the unloading is completed, the earthmoving machine enters the site. The rubbish is flattened and compacted to make the rubbish density more than 1 t/m^3^. Next, the most important step is to use a HDPE film with a width of 6 m and a thickness of 0.75 mm to temporarily cover the rubbish to prevent the odor which is emitted into the air, and rainwater seeps into the garbage dump. At this point, the landfill operation is basically completed. The leachate produced by the garbage pile is collected into the sewage storage tank through the crisscrossed high-density polyethylene pipes in the bottom of the reservoir, then it is processed at the landfill sewage treatment plant. Landfill gas (mainly composed of biogas) is transported to the biogas power plant for power generation through gas collection wells and pipelines and is integrated into the East China Power Grid. The biogas power plant can generate 280,000 kWh of electricity per day. Using the operation flow shown in Fig. 1, the average daily landfill operating area is 1800 m^2^. This study examines the change in the release of odors using NH_3_ and H_2_S as indicators before and after the coverage in order to characterize the effect of daily cover treatment on the reduction of odors. Among them, the release of odor after covering mainly examines the release amount of the membrane pores.

### Odor monitoring indicators and methods

#### Odor monitoring indicators

There are many types of odors in landfills, including saturated hydrocarbons, unsaturated hydrocarbons, organic alcohols, aromatic hydrocarbons, halides, sulfur compounds, mercaptans, and inorganic compounds (Lu et al. 2015; Sonibare et al. 2019). In this study, NH_3_ and H_2_S were used as indicators for the odor investigation based on the following three points: (1) Long-term monitoring results of the landfill showed that the gas concentrations of ammonia and hydrogen sulfide released by the landfill account for 84.05–93.94% of the total odor concentration (Ding et al. 2012). Ammonia and hydrogen sulfide are the main odor gas components emitted by the landfill. (2) Ammonia and hydrogen sulfide release reflects the degradation of nitrogen and sulfur in the waste dump (Ding et al. 2012; Asadollahfardi et al. 2019; Lim et al. 2018), while nitrogen and sulfur are the main constituent elements of fresh garbage, especially kitchen waste. NH_3_ and H_2_S can accurately reflect the odor generation on the mixed fresh landfill operation surface, and (3) the prolonged exposition to NH_3_ and H_2_S for a long time causes serious impact on health (Aatamila et al. 2010; Ding et al. 2012; Lee et al. 2006).

#### Odor collection point

Before and after the landfill day cover operation, 6 sampling points on the exposed working surface and film holes (8) on the temporary cover layer were randomly selected for odor collection and monitoring. The in situ gas collection method (Fig. 1) of the static box is used for odor collection, and the gas collection bag is a Tedlar gas collection bag with a volume of 0.5 L.

**Fig. 2:**
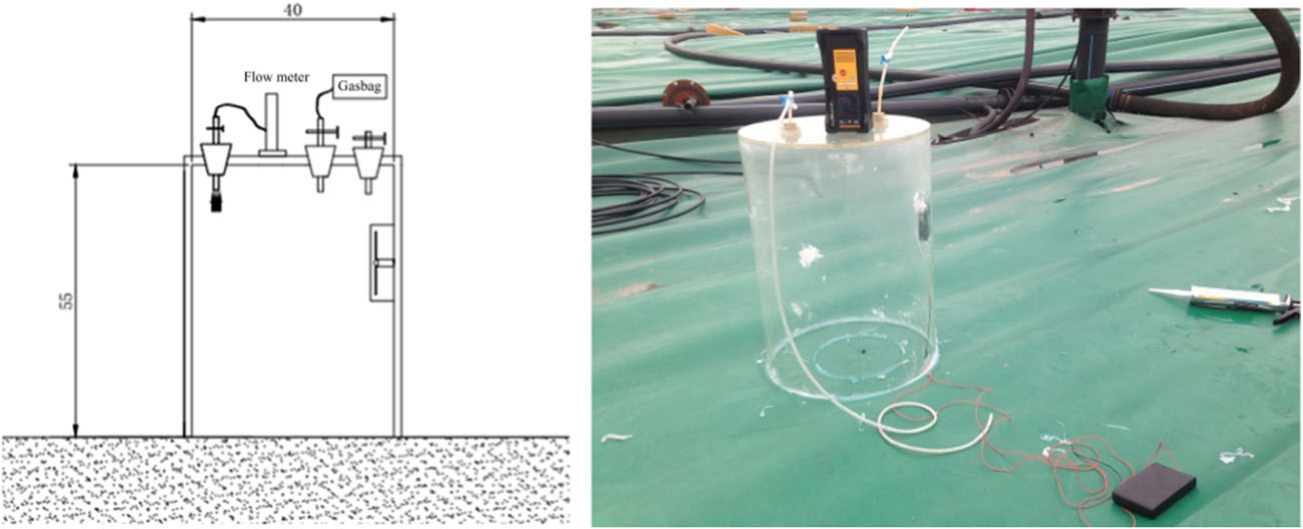
Large-size static box

**Fig. 3:**
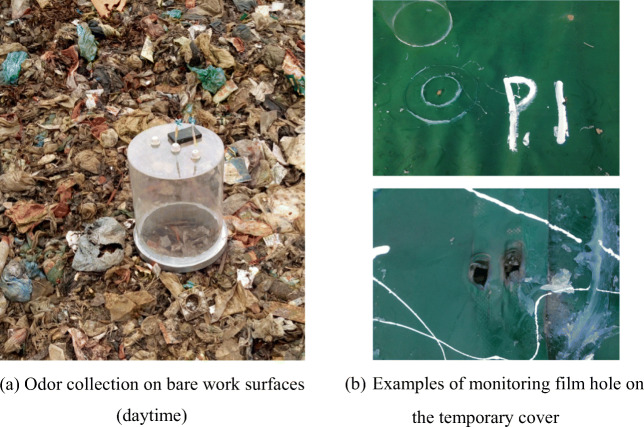
Example of on-site photos of landfill odor collection

On the exposed working area, considering the possible influence of day and night changes on gas release, a day was randomly selected in June, and day and night monitoring was conducted at 6 AM, 12 PM, 6 PM, and 12 PM throughout the day and 6 h (Fig. 2). On the temporary cover layer, the damage points (P1–P8, Fig. 2) were randomly selected for odor collection (Fig. 3). The gas monitoring time of the membrane pores was measured three times at 0, 15, and 30 min between 10:00 and noon.

#### Index analysis method

In order to examine the odor release from the membrane holes on the temporary cover and its effect on odor control, this study uses NH_3_ and H_2_S gas release fluxes for characterization. The gas release flux mainly uses the static box shown in Fig. 1 to collect odor at the gas collection points described in the “Odor collection point” section and then measures the concentration of hydrogen sulfide and ammonia, and then calculates the release flux according to the following formula.

At the *t* + Δ*t* moment, the mass of the gas to be measured in the box is the amount of *t* moment plus the amount of Δ*t* moment entering:

1 $$ \mathrm{V}{\mathrm{C}}_{t+\varDelta t}=\mathrm{V}{\mathrm{C}}_t+{j}^{\prime } s\varDelta t $$

2 $$ {j}^{\prime }=\frac{V}{S}\frac{\mathrm{dc}}{\mathrm{dt}} $$

where *V* is the box volume (m^3^), *C* is the target gas concentration (mg/m^3^), *S* is the box bottom area, and *j* is the gas flux (mg/m^2^/h).

To correct the flux in combination with ambient temperature, we use the following equation:

3 $$ j={j}^{\hbox{'}}\times \frac{273}{273+t} $$

Among them, the concentration of hydrogen sulfide and ammonia is mainly detected by spectrophotometry. The gaseous components are transferred to an aqueous solution by bubbling the gas in a sulfuric acid and zinc acetate solution, respectively. NH_3_ uses Nessler’s reagent spectrophotometry; methylene blue colorimetry was used to monitor H_2_S concentration (Ding et al. 2012).

### Estimating total odor release using linear modeling

A mathematical linear model was used to estimate the total amount of NH_3_ and H_2_S released from the exposed working surface and the temporary covering layer, and then used to evaluate the effect of the number of membrane holes on odor reduction. The model is based on the following assumptions: (1) study the odor reduction effect of the inner membrane pores within 24 h, (2) the landfill operation is carried out simultaneously with the temporary cover. The daily exposed operation area of the landfill is the same as the temporary coverage area, (3) ignoring the difference in odor emissions caused by the time difference between dumping, compaction, and temporary covering operations. The odor release time of bare operations is *t*, calculated based on the actual operation time of garbage dumping, and compaction and the gas release time in the temporary covering layer is 24*-t*. (4) We only consider the amount of odor released from the surface of the landfill, and we do not consider the actual environmental odors caused by factors such as air diffusion.

Based on the above assumptions, the total amount of NH_3_ and H_2_S released through the film holes on the exposed operation and the temporary cover layer is:

4 $$ {Y}_1=\left({Q}_{\mathrm{N}{\mathrm{H}}_3}+{Q}_{{\mathrm{H}}_2\mathrm{S}}\right)\times t\times 3.6\times A=3.6\mathrm{At}\left({Q}_{\mathrm{N}{\mathrm{H}}_3}+{Q}_{{\mathrm{H}}_2\mathrm{S}}\right) $$

5 $$ {Y}_2=\left({Q}_{\mathrm{N}{\mathrm{H}}_3}+{Q}_{{\mathrm{H}}_2\mathrm{S}}\right)\times \left(24-t\right)\times 3.6\times \mathrm{A}\div (100W)\times B=\frac{0.036\mathrm{AB}\left(24-\mathrm{t}\right)\left({Q}_{\mathrm{N}{\mathrm{H}}_3}+{Q}_{{\mathrm{H}}_2\mathrm{S}}\right)}{W} $$

6 $$ Y=Y1+Y2 $$

Among them, *Y* is the total amount of odors emitted from the operation area throughout the day; *Y*1 is the total amount of odors released in the working area without cover per day; *Y*2 is the total amount of odors released through the membrane holes on the daily temporary cover, g; QNH_3_ and QH_2_S are the release fluxes of NH_3_ and H_2_S on the exposed working surface and membrane hole, respectively, expressed in mg/(m^2^ s); *t* is the daily waste dumping and compaction operation time, that is, bare operation time, h; *A* is the landfill operation area, m^2^; *W* is the cover film width, in meters, the actual film width is usually 1–8 m; and *B* is the number of film holes per 100 m of film, *B* ≥ 0.

### Data processing method

Origin and EXCEL were used for data processing and charting, and SPSS 16.0 (SPSS for Windows, version 16.0, USA) was used for statistical tests (*p* < 0.05).

## Results and discussion

### Odor release flux on the exposed landfill surface

**Fig. 4:**
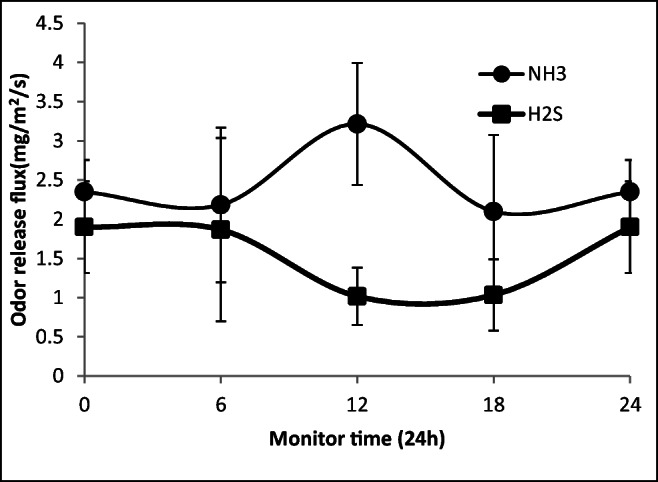
The average flux of odors release from the landfill operation area (6 sampling points, 0 and 24 in the figure are the same monitoring time)

The release fluxes of NH_3_ and H_2_S throughout the day in the 1–6 # monitoring points on the exposed working surface of the landfill operation area are shown in Fig. 4. The release flux of NH_3_ reached 2.1–3.2 mg/m^2^/s with an average of 2.5 ± 0.9 mg/m^2^/s, while H_2_S reached 1.0–1.9 mg/m^2^/s with an average of 1.5 ± 0.9 mg/m^2^/s. Our results show that waste in the exposed area releases NH_3_ and H_2_S in agreement with previous studies. The release rate of NH_3_ is 1.2–3.2 times higher than that of H_2_S. This is mainly due to the presence of aerobic and anaerobic zones in the landfill. At the surface, oxygen accelerates the ammoniation of organic nitrogen compounds (Ding et al. 2019; Lim et al. 2018). The release of H_2_S is mainly based on the anaerobic sulfate reduction by anaerobic microorganisms (mainly sulfate-reducing bacteria) (Asadollahfardi et al. 2019; Lee et al. 2006). However, since the odor threshold of H_2_S (1.5–150 g/m^3^) is much lower than that of ammonia (500–1000 g/m^3^) (Ding et al. 2012), the acute toxicity of H_2_S is much greater than that of ammonia. Occupational exposure is taken as reference. The human exposure to 76 ppm hydrogen sulfide for 10 min can cause death, while the required ammonia concentration leading to death is 2700 ppm within the same exposure time. This is 35.5 times that of H_2_S as recently reported (the United States Environmental Protection Agency 2020). Therefore, even though the NH_3_ release from the landfill surface has reached 3.2 times that of H_2_S, the discomfort caused by H_2_S and its impact on the health and environment are greater. This justifies why many documents list H_2_S as one of the main goals of odor control in the landfill (Ding et al. 2012; Lu et al. 2015; Aatamila et al. 2010). Therefore, the release of H_2_S with 1.0–1.9 mg/m^2^/s throughout the day should not be underestimated and should be of great concern to managers and operators.

The release of ammonia was seen to increase up to 3.2 ± 0.8 mg/m^2^/s at noon, then decreases and stabilizes between 2.1 and 2.3 mg/m^2^/s. This increase corresponds to the daily photoperiod and the relatively high temperature at 12 noon in this region. The high temperature enhances the ammoniation and the decomposition of complex compounds by the existing microorganisms more vigorous (Ding et al. 2019). Furthermore, the relatively high temperature accelerates the volatilization of ammonia (Lim et al. 2018), allowing higher ammonia release from the collection points. Surprisingly, the release of H_2_S does not increase within the photoperiod. H_2_S was higher in the dark period with 1.9 ± 1.2 mg/m^2^/s at 6:00 AM, then its concentration decreased during the day. Recently, the H_2_S release was studied to depend on the anaerobic microorganisms’ activity in the landfill, rather than the aerobic ones found at the landfill surface (Asadollahfardi et al. 2019; Lee et al. 2006). Besides, the metabolic activities of the microorganisms in the landfill generate local heat, allowing the temperature of the stack waste to be maintained at relatively stable values that are not affected throughout the day. This aspect is still open and needs deep investigation with respect to the kind waste, the depth, and the generated leachate.

### Odor release from membrane holes in temporary cover

**Fig. 5:**
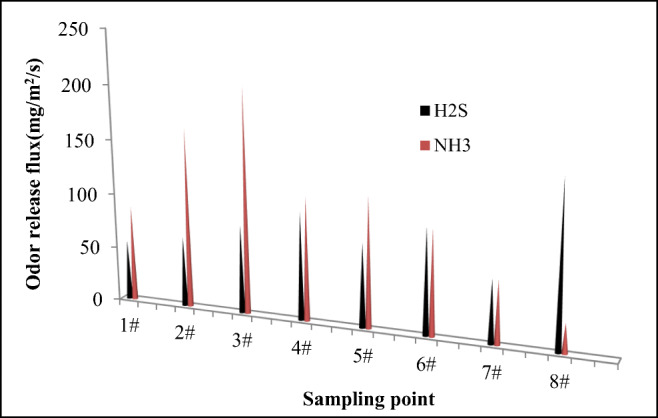
Gas release flux from the membrane holes on the HDPE cover (8 membrane holes, the average values are [NH_3_] = 109.6 ± 56.6 mg/m^2^/s; [H_2_S] = 86.0 ± 31.1 mg/m^2^/s).

In order to reduce the release of odors, temporary covers are often used in landfill operations. The studied landfill used a 0.75-mm HDPE film for temporary coverage. However, holes are easy to appear due to the settlement of the pile, construction, and characteristics of the landfill materials during the landfill operation. Due to the gas diffusion properties, odors escape from these membrane holes. Figure 5 shows the concentration of NH_3_ and H_2_S released from the membrane pores.

As shown in Fig. 5, the 8 randomly selected membrane pores all released NH_3_ and H_2_S with a certain flux. The NH_3_ concentration varied from 27.6 to 206.3 mg/m^2^/s and the H_2_S concentration 55.4–151.2 mg/m^2^/s, respectively. The average values were 109.6 ± 56.6 mg/m^2^/s and 86.0 ± 31.1 mg/m^2^/s. Such high released concentrations of NH_3_ and H_2_S from the membrane pores indicate that the landfill waste contains more degradable organic matter with proportions that can reach 50–70% as reported before (Zhou et al. 2014). These materials are degraded by specific microorganisms such as nitrifying bacteria (including ammonia-oxidizing bacteria, AOB) and sulfate-reducing bacteria. This aerobic–facultative–anaerobic microenvironment formed under the membrane, leading to the release of ammonia and hydrogen sulfide. The difference in the ammonia and hydrogen sulfide concentrations is mainly due to the difference in the activity of microorganisms that degrade nitrogen and sulfur.

Statistical analysis showed that among the 8 membrane holes randomly selected, there was a significant difference in the ammonia gas fluxes of 2 #, 3 #, 7 #, and 8 #, and the release of hydrogen sulfide from 1 #, 7 #, and 8 #. There is a significant difference in flux, which indicates that the odor emission is different between different membrane pores, which is mainly affected by factors such as membrane pore size and gas flow dynamics (Fox et al. 2011; Take et al. 2012; Rowe and Abdelatty 2012). This latter and its specific relevance need further study. However, although the NH_3_ and H_2_S emission fluxes of the 8 randomly selected membrane holes are different, they all emit NH_3_ and H_2_S. It can be seen that the membrane holes are important for the release of foul pollutants on the temporary cover and require great attention (McQuade and Needham 1999; Plaza et al. 2007).

Furthermore, compared with the release of NH_3_ and H_2_S on the exposed working surface (as presented in the “Odor release flux on the exposed landfill surface” section), the release of NH_3_ and H_2_S from the membrane pores of the cover layer is significantly larger. The average release concentrations of NH_3_ and H_2_S from the pores are respectively 43.8 and 57.3 times those of the exposed working surface. Such high concentrations of NH_3_ and H_2_S from the membrane pores are mainly due to the gas diffusion characteristics. The NH_3_ and H_2_S generated by the decomposition of organic substances under the membrane cannot be diluted and diffuse quickly to the air due to the insulation of the cover layer. Nevertheless, these gasses concentrate just at the membrane holes before release. Overall, the HDPE coverage does reduce the large-area leakage of NH_3_ and H_2_S odors (Plaza et al. 2007), but the existence of the holes intensified the diffusion flux of “point” sources of odors’ dispersion.

### Effect of film pores on odor reduction effect

As shown previously in this study, the presence of membrane pores allows the NH_3_ and H_2_S to be released at high concentrations on the temporary cover layer. If the appearance of film holes can be prevented, it is undoubtedly an effective way to control odors, but it is ideal and difficult to avoid the appearance of film holes on the temporary cover layer during actual operation (Gilson-Beck 2019; McQuade and Needham, 1999). Therefore, researchers have carried out a lot of research on the occurrence probability of membrane holes (Nosko and Crowther 2015), quantification methods (Take et al., 2007), and their repair technologies such as leakage location technology (Gilson-Beck 2019). The film holes on the cover layer are closely related to the effect of landfill odor control, and the daily cover of HDPE film is not cheap. At present, the cost of covering HDPE film in China is about 100,000 RMB (approximately $ 14,900) per hectare (Chen et al. 2011). Therefore, the number of membrane holes will directly determine whether the economically expensive behavior of temporarily covering HDPE is worthwhile for odor control. The threshold of the number of membrane holes needs to be further clarified to provide a reference for actual project management and operation. Next, based on the obtained results and the actual status of the actual landfills investigated, we showed the effect of the number of membrane holes on the odor reduction effect.

Based on the total odor release estimation model established in the “Effect of film pores on odor reduction effect” section, the daily average operating area “*A*” of the investigated landfill in this study was 1800 m^2^, and the actual bare operation time “*t*” was 6 h. Accordingly, the average releases of NH_3_ and H_2_S were calculated using Eq. 4. The total release on its exposed working surface can be calculated as:

*Y*1 = 3.6 *At* (*Q*_NH3_+*Q*_H2S_) = 3.6A*t*(2.5 + 1.5) = 14.4 *At* = 86.4*A* = 1.56*10^5^ g = 156 (kg)

The landfill is in accordance with the operation flow of Fig. 1. After the waste pile is compacted, HDPE with a 6-m width is used for daily coverage after welding. From Eq. 5, the total odor released from the film holes on the temporary cover layer in the remaining (24-*t*) hours can be obtained as:

$$ {Y}_2=\frac{0.036\mathrm{AB}\ \left(24-t\right)\left({Q}_{\mathrm{N}{\mathrm{H}}_3}+{Q}_{{\mathrm{H}}_2\mathrm{S}}\right)}{W}=\frac{0.036\mathrm{AB}\ast \left(24-t\right)\left(109.6+86\right)}{W}=\frac{7.04\mathrm{AB}\left(24-t\right)}{W}=\frac{7.04\mathrm{AB}\ast \left(24-6\right)}{6}=21.1\mathrm{AB}=37.98B\ \left(\mathrm{kg}\right) $$

**Fig. 6:**
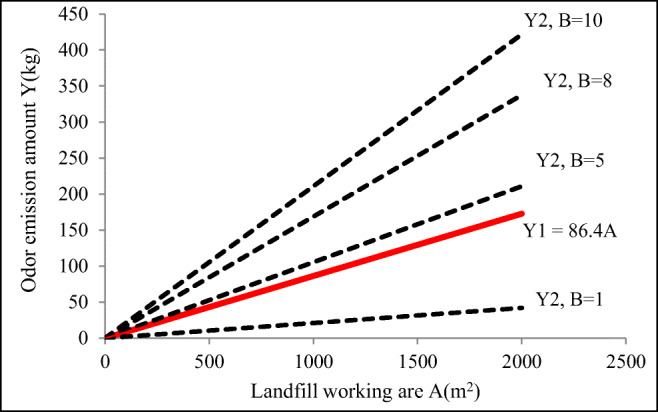
Mathematical modeling of the total amount of odors release as a function of the landfill working area

In the above *Y*2, *B* is the number of film holes per hundred meters of film, and *B* ≥ 0. Based on the above two equations, it can be concluded that the relationship between *Y*1, *Y*2, and the working area is shown in Fig. 6, where *Y*2 and *B* value are closely related.

Figure 6 shows that the total amount of odor emission increases with the increase of landfill operation area with a linear relationship (Liu et al. 2015). In addition, the *Y*2 released on the daily cover layer fluctuated above and below *Y*1 as the *B* value changed. It can be seen that the total amount of odors released by the film holes from the cover layer depends on the landfill operation area (that is, the coverage area), which has a close relationship with the number of film holes. In the case of a certain landfill operation area, the number of film holes in the cover layer will determine whether the temporary cover operation has the effect of reducing odors.

Furthermore, Eq. 6 is used to estimate the total amount of odors *Y* and *Y*′ released under the two scenarios (with and without covering) as follows:

$$ {\displaystyle \begin{array}{l}\kern4.5em Y={Y}_1+{Y}_2=14.4A{t}_1+\frac{7.04\mathrm{AB}\left(24-{t}_1\right)}{W}=86.4A+21.1\mathrm{AB}\\ {}Y^{\prime }=Y1=3.6\ {At}_2\left({Q}_{\mathrm{NH}3}+{Q}_{\mathrm{H}2\mathrm{S}}\right)=14.4\ {At}_2\end{array}} $$

In order to obtain the maximum threshold value of the number of film holes, it can be known from the above formula that assuming that the temporary film deodorization operation of the landfill is meaningful, then *Y* < *Y*′. That is, 14.4 *At*1 + 7.04AB * (24-*t*1)/*W* < 14.4*At*2, i.e.,

$$ B<\frac{14.4W\left({t}_2-{t}_1\right)}{7.04\left(24-{t}_1\right)} $$

In this equation, according to the scenario assumption, *t*_2_ is the exposure time when the covering operation is not adopted. It is 24 h according to the first assumption (see the “Effect of film pores on odor reduction effect” section). When *t*_2_ is brought into the above formula, we can know that if *Y* < *Y*′, then *B* < 2.05*W*.

From the above analysis, the maximum number of film holes allowed on the film is determined only by the film width (*W*) and has nothing to do with the exposed working time (*t*) and the working area (*A*). It can be found that from the perspective of odor reduction, the larger the film width, the more film holes are allowed as shown in Table 1.

**Table 1:** The maximum number of membrane holes allowed under different film widths

Film width (m)	1	2	4	6	8
Max holes allowed	2	4	8	12	16

Therefore, in the actual landfill treatment, the appropriate film width should be selected based on the mechanical operation level while designing, welding, and constructing (McQuade and Needham, 1999). Taking the landfill investigated in this study using a 6-m-wide HDPE film for temporary coverage as an example, it is recommended that the number of membrane holes per hundred meters of the landfill site should be ≤ 12; otherwise, the effect of reducing odor will not be exerted. When the number of film holes within 100 m is 8, the total amount of odor released is 73.8% of the bare operation, and the amount of odor reduction reaches 26.2%. It can be seen that from the perspective of the total amount of odor release, the daily cover film can effectively reduce total odor emissions if the optimal hole number is respected. At this level, it is worth to mention that the covering operation itself may generate odor emissions.

In the actual landfill operation, in order to reduce welding and facilitate the process, a film more than 4-m wide is usually selected for daily coverage. According to Table 1, the permissible number of pores in the 100-m-long inner membrane is 8. In the actual operation process, if the construction method is correctly standardized and the occurrence of unexpected events such as the fall of tools is minimized as possible, the number of film holes in the film covering operation can be controlled within this level range. Hence, the use of daily mulching operations in actual engineering is still significant for reducing the total amount of odors.

## Conclusion

In this study, the Hangzhou landfill operating at full load was used to study odors release that can be harmful for the neighboring environment. The released odors’ concentration of NH_3_ and H_2_S from the film holes on the exposed working surface of the landfill and its daily cover were quantified and compared to understand the effect of the film holes. We found that the film holes on the cover of the daily operation in the landfill released higher concentrations of NH_3_ and H_2_S, which are 43.8 and 57.3 times the average release in the exposed working area, with NH_3_ being 2.5 ± 0.9 mg/m^2^/s and H_2_S being 1.5 ± 0.9 mg/m^2^/s. Although the daily film coverage of the landfill reduces the large-area escape of NH_3_ and H_2_S odors, the existence of membrane holes has increased the diffusion of odors forming “point” sources. A linear model of the total odor emission of NH_3_ and H_2_S is used to evaluate the effect of the number of membrane holes on the odor control of the landfill. We found that the total amount of odor emission on the exposed working surface and the temporary covering layer showed a linear positive correlation with working area. In order to ensure the deodorizing effect of the covering operation, there is a maximum allowable film hole number threshold on the covering layer, which is independent of the working area and exposed working time, and is mainly determined by the HDPE film width (*W*). For the landfill in this study, a 6-m-wide HDPE film was used for daily coverage. If the number of membrane holes within 100 m is less than 12, the daily coverage operation is meaningful for odor control. If the number of membrane holes within 100 m of the landfill is only 8, the total reduction of odor emissions can reach 26.2%.

In this study, by comparing the NH_3_ and H_2_S odors release from the exposed operating surface and the film holes in the cover layer of landfill, the effect of film holes on the cover layer on the odor emission of the landfill was discussed. From the perspective of controlling the total odor emissions, we clarified the threshold of the number of film holes on the daily cover and its impact by modeling and provided an important reference for the design, operation, and decision-making of the daily cover operation of the landfill. It should be noted that the mathematical linear model used in this study is relatively rough and simple, and should be further optimized based on the characteristics of the landfill, such as landfill age, membrane hole size, and weather condition. In addition, the cause of the membrane hole analysis, the composition of released odor, and long-term release law can be further improved in future work.

## Data Availability

All data generated or analyzed during this study are included in this published article.
